# Breakfast skipping, late dinner intake and chronotype (eveningness-morningness) among medical students in Tabuk City, Saudi Arabia

**DOI:** 10.11604/pamj.2019.34.178.16250

**Published:** 2019-12-05

**Authors:** Hyder Osman Mirghani, Khalid Saleh Albalawi, Omar Yarub Alali, Waled Mohammed Albalawi, Khalid Mohammed Albalawi, Talal Rabea Aljohani, Wedyan Saleh Albalawi

**Affiliations:** 1 Faculty of Medicine, University of Tabuk, Saudi Arabia

**Keywords:** Breakfast skipping, late dinner intake, chronotype, academic performance, medical students

## Abstract

**Introduction:**

There is an increasing awareness regarding meal timing and chronotype. The present study aimed to assess breakfast skipping, late dinner intake, and chronotype among Saudi medical students.

**Methods:**

A cross-sectional study was conducted among 169 clinical phase medical students during the period from January to May 2017. A self-administered questionnaire was used to report the frequency and timing of breakfast and dinner. In addition, the previous cumulative grade average, bedtime, wake-up time, and sleep duration during working days and weekends were reported. The chronotype was calculated from mid-sleep and wakeup time during weekends and sleep dept. The student's weight and height were measured to assess the body mass index (BMI). Participants also completed a diary detailing their sleep habits for two weeks before filling out the questionnaire. The chi-square and Pearson's correlation were used for the statistical analysis.

**Results:**

Out of 169 medical students (48.5% males), their age was 22.90±1.27 years, 42% were breakfast-skippers, while 49.7% were late dinner consumers. No correlation was found between the previous cumulative grades (GPA), BMI, chronotype, and time lag in wakeup and bedtime between weekdays and weekends (p>0.005). No significant statistical differences between breakfast-skippers and late dinner consumers and their counterparts regarding GPA and chronotype.

**Conclusion:**

Breakfast skipping and late dinner consumption were prevalent among medical students in Tabuk, Saudi Arabia, future large sample case-control studies to assess the impact of meal timing, and chronotype on academic performance are highly recommended.

## Introduction

Sleep is essential for various body vital functions including memory, and preservation. Healthy sleep behavior is critical for immune response modulation and clearing of the waste products from the brain [1]. Neural and environmental factors extensively regulate the sleep-wake cycle. People differ in eveningness-morningness (chronotype) which is the circadian phase preference. Furthermore, the social obligation could substantially alter the natural sleep clock (people are forced to stay awake or sleep against their chronotype leading to circadian misalignment) leading to social jet lag [2]. Persons with a trend towards rising early in the morning, perform mentally and physically best in the morning hours and go to bed early in the evening are morning chronotype (larks), while those with a tendency to rise later in the morning, stay awake later at night, and perform at their best in the late afternoon or evening are evening chronotype (night owls). These preferences are assumed to have unique, genetic, biological, contextual, and psychosocial components. Diurnal preferences had been linked to various habitual and non-habitual issues like eating habits, sleeping behavior among University students, smoking, and drug use [3]. Breakfast is often thought of as the principal meal of the day. It significantly contributes to the daily energy requirement and improves cognition [4]. Eating a late dinner could lead to breakfast skipping. Furthermore, eating pattern seems to follow the internal clock rather than socially determined. Thus late chronotype is associated with a shift of meal consumption toward a later time of the day leading to breakfast skipping and late dinner intake [5]. There is an increasing awareness regarding the lethal effects of meal timing and chronotype on physical and mental well-being. Few researchers have studied the meal pattern and chronotype among medical students in Saudi Arabia. Thus, we conducted this survey to assess the breakfast skipping, late dinner consumption, and chronotype among medical students in Tabuk, Saudi Arabia.

## Methods

A cross-sectional descriptive study conducted to assess the breakfast skipping, late dinner intake, and chronotype among the clinical phase medical students in Tabuk City Saudi Arabia during the period from January to May 2017.

**Study areas and setting:** the Medical College, University of Tabuk.

**Sample size and technique:** all the clinical phase medical students (in the 4^th^, 5^th^ and 6^th^ classes) were approached, total number 220 (response rate 76.8%).

**Study variables and data collection tools:** the following data were collected age, sex, breakfast skipping, late dinner intake, weekdays and weekend bedtime and wake up, the sleep duration during the weekdays and weekends, and the lag in hours between weekend and weekend's bedtime and wake up. The mid-sleep time was estimated as the midpoint between sleep onset (bedtime plus sleep latency) and wake time. The weight to the nearest 0.1kg and height to the nearest 0.1cm were measured to calculate the body mass index (BMI). For this research the following definitions were adopted: i) The chronotype: the mid-sleep time on free days (weekends) subtracting 0.5 the sleep debt. ii) Sleep debt: the sum of the weekend sleep duration (the length of sleep onset time until wake time) minus the weekly average sleep duration [6]. iii) Breakfast skipping: breakfast was defined as any food or beverages consumed between 5:00 a.m. and 10:00 a.m [7], those who skipped breakfast for at least three days were defined as breakfast skippers. iv) Late dinner intake: dinner intake within 2 hours before bedtime at least three times per week were regarded as late dinner consumers [8]. v) The body mass index (BMI) was calculated using the formula: BMI=Weight (in kg) / Height (in meters squared). vi) cumulative grade average (GPA): the previous GPA.

**Data analysis:** the data were exported to the Statistical Package for Social Sciences for the analysis. The t-test and Pearson correlation were used to compare breakfast-skippers and late dinner consumers to their counterparts and to test the previous GPA correlation to chronotype and meal pattern. A P-value of <0.05 was considered significant.

**Ethical consideration:** approval was obtained from the Research Committee of the Faculty of Medicine, University of Tabuk. Written informed consent was obtained from all the participants.

## Results

Out of 169 medical students, (51.5% were females), 36.1 were from the 6^th^ class, 33.1% were from the 5^th^ class, while the fourth class medical students constituted 30.8%. In the present study, breakfast skipping was found in 42% and late dinner intake was reported in 49.7% of students (Table 1). In the current study, the participant's mean age was 22.90±1.27 years. The previous cumulative grade average was 3.63±0.66, the BMI was 25.01±5.20, the working days wake-up time was 6.12±2.58, while it was 10.56±3.03 for weekends. The bedtime was 23.33±5.17 and 25.63±2.39 for weekdays and weekends respectively, lag of wakeup time between weekdays and weekends was 4.46±2.12, lag of bedtime between weekdays and weekends was 1.87±1.85 and the mean chronotype was 6.19±1.91. The above data implied that medical students in Medical College, University of Tabuk were more likely of evening chronotype (Table 2). The current data showed no association between the cumulative grade average, chronotype, BMI, time lag in wakeup time and bedtime between weekdays and weekends, P-values=0.675, 0.376, 0.136, and 0.962 respectively (Table 3).

**Table 1:** Sex, class, breakfast skipping, and late dinner intake among the study group

Character	No%
**Sex**	
Males	82 (48.5%)
females	87 (51.5%)
**Class**	
4^th^ year	52 (30.8%)
5^th^ year	56 (33.1%)
6^th^ year	61 (36.1%)
Breakfast skipping	71 (42%)
Late dinner intake	84 (49.7%)

**Table 2:** Characteristics of the study sample

Character	Mean±SD
Age	22.90±1.27
GPA	3.63±0.66
BMI	25.01±5.20
Weekdays Bedtime	23.33±5.17
Weekdays Wakeup	6.12±2.58
Weekends Bedtime	25.63±2.39
Weekends Wakeup	10.56±3.03
Chronotype	6.19±1.91
The lag of wakeup time between weekdays and weekends	4.46±2.12
The lag of bedtime between weekdays and weekends	1.87±1.85

**Table 3:** Correlation of GPA to chronotype and the lag of time between weekdays and weekends wake-up and bedtime and body mass index

Character	Correlation	P-value
Chronotype	0.034	0.675
BMI	-.071	0.376
The time lag in wakeup time between weekdays and weekends	0.125	0.136
The time lag in bedtime time between weekdays and weekends	-0.004	0.962

## Discussion

In the present study, 42% of medical students were breakfast-skippers and nearly half-consumed dinner within two hours of sleep. No significant differences between breakfast-skippers and late dinner consumers regarding the previous GPA and chronotype (data not shown). No direct correlation was evident between the current GPA, chronotype, BMI, time lag in wakeup time between weekdays and weekends. The current data showed that 42% of clinical phase medical students were breakfast skippers in line with Sun *et al*. [9] who reported a prevalence of 41.7% among medical students in China. The present finding of the tendency towards eveningness in the current study were in line with our previous observations [10]. Late chronotype has been linked to health impairing lifestyles and poor quality of life [11]. A recent study published by Akram *et al*. [12] concluded that: no association between chronotype and cumulative grade point average, but late chronotypes were more likely involved in superficial learning. These findings were in line with the current observation in which no association was found between GPA and chronotype. Another study [13] showed that late chronotype was more likely not attending their class, and their performance in the examination was related to the examination time (poor performance when examined early in the day. In addition, the researchers found no relationship between chronotype and crystallized intelligence (humanistic/linguistic) supporting the current findings. The present data found no correlation between academic achievement and the time lag in wakeup time and bedtime between weekdays and weekends. Similarly, Harazsty *et al*. [14] observed no significant association between academic performance and sleep debt on workdays. A plausible explanation is that the performance is primarily affected by the examination time (the morningness students performed well in the morning and early afternoon, while the eveningness students were better in the late afternoon). Also, the lag of time between weekends and weekdays affected the performance in weekly tests rather than examinations periods. The current study showed no significant differences between breakfast-skippers and late dinner consumers regarding the GPA and chronotype in line with Roßbach *et al*. [5] who observed no correlations between chronotype, breakfast time, and lunchtime, these results could be explained in part by the meal contents because the chronotype, social jetlag, and perceived sleep debt could influence the type and amount of some food groups consumed at mealtimes [15]. Previous literature [16] observed that having a frequent intake of unhealthy meals negatively affects academic performance and not merely skipping meals. The present findings showed no association between breakfast skipping and the cumulative grades average supporting the previous observations. The results of this study should be viewed in the face of the following limitations: the fact that the study was conducted at a single college, the reliance on a self-reported questionnaire which is more prone to subjectivity, and the relatively small sample size. The major limitations of the current survey are the recording of the previous GPA which may not reflect the current situation and not controlling for various confounders that may affect the GPA.

## Conclusion

Breakfast skipping and late dinner consumption were prevalent among medical students in Tabuk, Saudi Arabia who were more likely evening chronotype, no significant differences between breakfast-skippers and late dinner consumers regarding the GPA and chronotype. Future large sample case-control studies to assess the impact of meal timing and chronotype on academic performance are highly recommended.

### What is known about this topic

- Chronotype is associated with breakfast skipping;
- Chronotype is associated with obesity.

### What this study adds

- Eveningness chronotype, breakfast skipping, and late dinner intake were high; among medical students which may put them at a high risk of health impairing behavior and impaired quality of life;
- The current data can be used as a base to guide future studies investigating the associations of late chronotype and meal timing on physical and mental health among university students.

## Competing interests

The authors declare no competing interests.
